# Beyond technology acceptance—a focused ethnography on the implementation, acceptance and use of new nursing technology in a German hospital

**DOI:** 10.3389/fdgth.2024.1330988

**Published:** 2024-04-25

**Authors:** Ronny Klawunn, Urs-Vito Albrecht, Deliah Katzmarzyk, Marie-Luise Dierks

**Affiliations:** ^1^ Department for Patient Orientation and Health Education, Institute for Epidemiology, Social Medicine and Health System Research, Hannover Medical School (MHH), Hanover, Germany; ^2^ Department of Digital Medicine, Medical Faculty OWL, Bielefeld University, Bielefeld, Germany

**Keywords:** ethnography, technology acceptance, UTAUT, participant observation, nursing care, implementation, technical innovations, Germany

## Abstract

**Introduction:**

Hospitalised patients could benefit from the emergence of novel technologies for nursing care. There are numerous technical products available, but these rarely find their way into practice. Further knowledge is required about the circumstances under which technology in nursing is accepted and used. In the research project “Centre for Implementing Nursing Care Innovations”, technical innovations are implemented on a trauma surgery inpatient ward in Germany. After implementation, it was investigated: Which implemented technologies are accepted/rejected, and which factors influence the acceptance/rejection of technology for nurses?

**Material and methods:**

A focused ethnography was used, containing two approaches: *First*, participant observation was conducted to examine nurses’ and patients’ interaction with technologies. Observations were fixed in a field research diary and analysed using evaluative qualitative content analysis. *Second*, a questionnaire was used by nurses to provide information about the use frequency and technology suitability. The results of the study were consolidated and analysed using the UTAUT model.

**Results:**

*S*even studied technologies can be summarised in four result categories: (1) A Mobilising mattress, a Special projector and a Sound pillow are accepted and used by nurses and patients, because they offer a way to provide high quality care with little additional effort. (2) A Fall prevention system is consistently used in patient care as a work obligation, but since nurses consider the system error-prone, acceptance is low. (3) An Interactive therapy ball is accepted but nurses cannot use it due to the high workload. (4) An App for nurse-patient communication and a work-equipment tracking system are not used or accepted because nurses do not see a practical benefit in the systems.

**Discussion:**

Acceptance or rejection of a product does not necessarily equate to use or non-use of the technology. Before implementation, technology acceptance among users occurs as prejudice—when users are given time to experiment with technology, intention-to-use can stabilize into sustained use. Accepted and used technologies can serve to mask problems (such as staff shortages) and encourage problematic developments, such as the reduction of contact time at the bedside. Therefore, technology acceptance should be qualified in asking *to what* accepted technology contributes.

## 1 Background

### 1.1 Nursing, technology and acceptance

With the growing use of digital technologies in healthcare, new technologies become increasingly available for nursing in recent years. For this profession in particular, technology is one possible response to the challenges of an ageing population being cared for by a decreasing number of available professionals (1, 2). Technology uptake in nursing care needs to accelerate to use the potential benefits of new technologies, and enablers and barriers related to technology implementation should be investigated and understood. Potential factors are numerous, e.g., a lack of fit between technology output and user need, inappropriate design for use needs, misguided implementation efforts or institutional limitations (3, 4). These could have an impact on the use and acceptance of nursing technologies.

Behavioural intention *or* actual use of technology has been studied regarding the acceptance of nursing technology. However, while new technology is implemented, the user's perspective may change due to the occurrence of unintended or unanticipated consequences of technology use (5), the social and contextual influences of implementation or facilitating conditions. For instance, through getting to know a new device and getting used to its functions and abilities, a negative expected usefulness and ease of use may shift to a positive attitude and vice versa. More research is needed to learn how and why behavioural intention shifts to a sustained and accepted actual use or a disruption of use and rejection of technology.

### 1.2 State of research

The adoption of new technologies in nursing is related to various determinants of technology acceptance. In the case of tele-nursing and remote visual monitoring of patients, studies have indicated that while the technology may reduce the number of falls, the acceptance of technology may only be moderate (6). Similarly, in the case of mobile healthcare communication tools, it has been shown that promoting early adopters can significantly influence user's behavioural intention to use the technology (7, 8). Similarly, in the case of mobile healthcare communication tools, it has been shown that promoting early adopters can significantly influence user's behavioural intention to use the technology (9). Users tend to favour mobile tools for inter-professional or professional-patient communication when tools are easy to use and efficient (10, 11). For AI technology that improves decision-making, another study have found that technology acceptance may be high among nurses and other professionals if the technology incorporates professional expertise and evidence into decision-making (3). However, such a technology may be associated with fears of loss of autonomy and expected negative impact on clinical workflows (12).

Only some studies have investigated how and why the intention to use technology in nursing may shift towards accepting or rejecting it after implementation. One study in a critical care nursing unit has demonstrated in a pre/post comparison of technology implementation that self-concern and expectation for ease of use decreased for nurses after adapting the technology (13). However, concerns about technology's impact on practice and perceived usefulness increased at the same time (ibid.). Another study has investigated the implementation of a digital oral healthcare intervention in Norway. As users adopted the new technology, they gradually changed their mode of use from—what the authors described as—“norm-based to routine-based behaviour”, highlighting the relevance of familiarisation with technology and the corresponding shift of user behaviour (14). For tele-nursing technology, it has been shown that only the performance expectancy was significant for caregivers’ behavioural intentions. After introducing the technology, the facilitating conditions and the performance became relevant for caregivers (15).

### 1.3 Research project and research question

The “*Centre for Implementing Nursing Care Innovations*” study (Funding: German Federal Ministry of Education and Research, funding number 16SV7892K) aims to implement new technologies in a trauma surgery inpatient ward of a university hospital in Germany. After technology introduction, we investigate the modes nurses’ use technologies and how patient care and nursing processes will change during technology implementation. The research question is:

*Which implemented technologies are accepted/rejected by nurses, and which factors influence the acceptance/rejection of these technologies?*

We conducted an ethnographic study and evaluated and reported the results using the *Unified Theory of Acceptance and Use of Technology 1*-model (16). The advantage of this model, which unifies eight separate models, is the provision of various explanatory factors that can predict or explain both the intention to use technology and the actual use (17). UTAUT conceptualises acceptance and use not merely as individual user decisions but places user behaviour and intentions in the context of institutional, organisational, and social environmental factors that may be influenced by mediating factors (age, gender, experience and voluntariness of use).

The study's implementation strategy allows to investigate how behavioural intention to use technology may shift to actual acceptance or rejection. Following Greenhalgh et al., this strategy involves two approaches: (1) We cooperated with the study hospital and managerial nursing staff to create institutional conditions for a successful and sustainable introduction of new technology to facilitate change of working structures (implementation) (18). (2) To select suitable technologies, we involved nurses from the study ward in a participatory manner by consulting them about potential technology and its usefulness (dissemination) (ibid.). For this purpose, we identified areas of nursing care on the project ward that could be supported with technical solutions—these areas involved, for instance, assistance with geriatric patients, dangers related to falls or pressure ulcers, inefficient patient communication or long walking distances (19). Based on these areas of need, the research project first took a closer look at potentially useful technologies and examined their implementability. For this purpose, an internal guideline was developed that included the IT perspective, nursing science, ethical, legal and social implications and the known study literature on the technology (19, 20). Once the potential technical and organizational implementability of the technology had been confirmed, it was presented to nursing staff. In workshops, they reflected on their behavioural intention to use the technology within their daily working routine (21, 22). If nurses showed their interest in using the presented technology and therefore articulated their intention to use it, the implementation of the product followed. Afterwards, the use of technology and patterns of acceptance or rejection has been observed. All costs that are associated with the purchase and maintenance of the technology were covered by the project budget as part of the research project. In the case of maintenance and repair work, the corresponding effort was shared between employees of the project station and the research project (see Limitations).

### 1.4 Overview of implemented technology in the research project

During the research and implementation activities, seven technologies were implemented and researched at the project ward, the technologies can be found in Table 1.

**Table 1:** List of implemented and studies technologies.

Short description	Information on technical integration
Technology for fall and pressure ulcer prevention	
1. An automated mobilisation mattress system that repositions patients in the bed to prevent pressure ulcers. ▪ Active Mobilisation System, Compliant Concept	The device runs autonomously from other technical systems, it requires a power connection. To document the use of the mattress, a checkbox was integrated into the patient documentation system. It collects usage data (frequency and duration of use) which, however, can only be retrieved by the manufacturer on site in the event of troubleshooting.
2. An automated fall prevention system that uses the nurses’ call light to send an alarm in case of patient bed exits. ▪ SafeSense Bed Exit System, Wissner Bosserhoff	The device connects to the nurse call system in the hospital ward via a cable connection. This is a closed system; the system merely registers a bed exit impulses. There is no automatic forwarding and documentation to other systems. It does not collect any data.
Technology for patients with challenging behaviour	
3. An audio-haptic sound pillow that plays atmospheric sounds and uses vibration to calm patients with dementia, agitation or restlessness. ▪ inmuRELAX, inmutouch	The system is powered by a rechargeable battery. It operates autonomously, there is no connection to other technical systems. It does not collect any data.
4. A special projector/beamer designed for health care institutions to calm or activate patients with dementia, agitation and restlessness. ▪ Qwiek.up, Qwiek	The system is powered via a socket. It operates autonomously, there is no connection to other technical systems. It collects usage data (frequency and duration of use) which, however, can only be retrieved by the manufacturer on site in the event of troubleshooting.
5. An interactive therapy ball that helps to activate patients or stimulate memories in patients with dementia. ▪ ichó therapy system, icho systems	The system is powered by a rechargeable battery. It operates autonomously, there is no connection to other technical systems. It does not collect any data.
Technology for improvement of communication and organisation during nursing care	
6. A Patient-Nurse communication app to facilitate communication between nurses and patients and assists nurses in organising and prioritising work processes. ▪ Cliniserve CARE, Cliniserve	The app is operated by the manufacturer via an external server in compliance with European data protection regulations. There is no connection to internal hospital IT systems. Accordingly, there is no automatic transfer of information for documentation purposes. Usage data is stored anonymously (without reference to the separate patient). Patients consent to the use of the app via the clinic’s data protection regulations. To use the app, patients can access mobile data or a Wi-Fi connection for patients and nurses can access a Wi-Fi connection for staff.
7. A webpage-based tracking system to locate work-related equipment on the project ward. ▪ Tracking System HYPROS TTI, HYPROS	The location information is sent via Bluetooth beacons to Wi-Fi hotspots. The location data is displayed and evaluated via a separate website. This website is password-protected and has no connections to internal hospital IT systems. There is no automatic transfer of information for documentation purposes.

## 2 Materials and methods

### 2.1 Study design

The study used a focused ethnographic, multi-methods investigation with a distinct qualitative emphasis. Focused ethnography is suitable for investigating social fields with high degrees of professionalism and functional differentiation by studying the entanglements and interactions between individual actors, institutional processes, settings and technologies (23). A main goal is to investigate social and cultural processes that are implicit or difficult to articulate for those being studied (24, 25). Compared to anthropological ethnography, the focused account is characterised by short field stays and an intense data collection phase (26). The following methods were applied:

1. **Participant observation** of nursing workflows to explore the use of implemented technologies and
2. **Questionnaire survey** to explore the nurses’ perspective on the usability of the implemented products.

**Figure 1:**
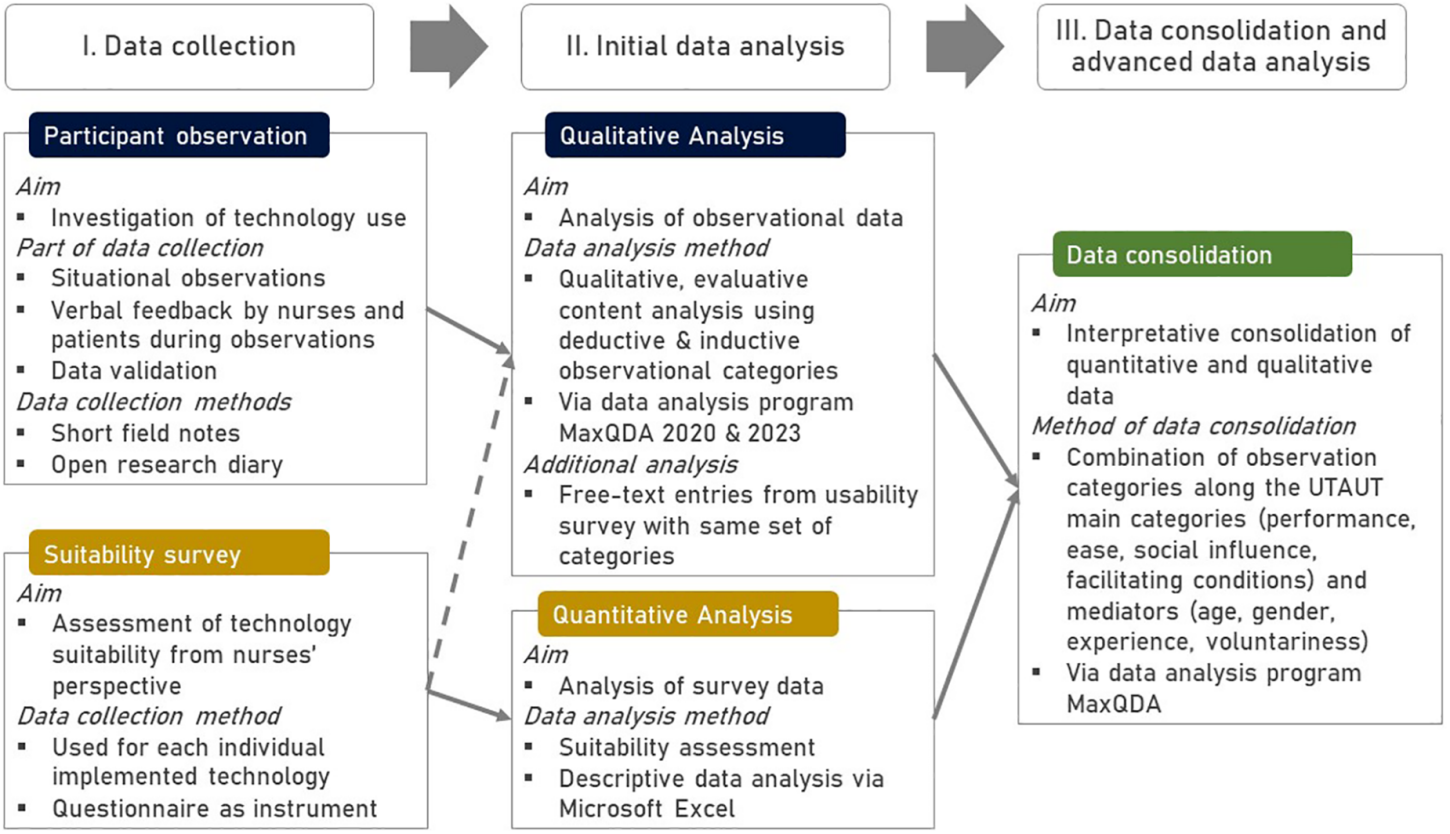
Research Design.

The steps of data collection, processing and analysis are described in the following sections, an overview of the research design can be found in Figure 1.

### 2.2 Methods 1: data collection

(a) Participant observation

The observation aimed to follow professional nurses during their workday for several hours to explore work processes and interactions with patients and other nurses with the introduced technologies. The observation was carried out by the author RK and conducted as an “observer as participant”, which means that the observer role tends to be passive, yet transparent to all participants in the field. The choice of non-functional, everyday clothing and a restrained accompaniment was intended to keep the observer passively in the background while enabling the investigation of a native perspective of the observed concerning specific “situations, activities and actions” (26).

One of the members of the research project (not the observer and no co-author) acted as a gatekeeper to gain access to the field, as he also worked as a nursing professional on the project ward. In the course of the observations, it was possible to establish personal relationships with other nursing staff who allowed access to the ward to observe shifts. In terms of recruitment, all nurses were eligible to participate whom: (1) worked as professionally trained on the project ward, (2) were currently using a technology of interest, (3) would like to be accompanied and (4) gave written consent to be observed (see Ethical Considerations section).

At the beginning, fixed time points for the observations were set. However, this pattern needed to be adjusted, e.g., because some technology was not used for an extended period and then used intensively for a short period. These required spontaneous station visits outside the fixed observation pattern until sufficient information for each technology was gathers. Another way of achieving data saturation was to present and discuss the results with the nursing staff on the ward (see section Quality Assurance).

An observation guideline (see Table 2) with specific questions was designed to help the observer during the field stay (27). These questions were developed deductively from existing models on technology implementation (28), adoption (29, 30), technology acceptance (16) and intention (31)—the guideline is shown in Table 2. The instrument was developed based on multiple theoretical starting points to integrate different perspectives on technology use. The categories were later integrated into the UTUAT model, which can also be found in Table 2 (see also *2.4,* *Methods III*). The guiding questions were discussed by the research team and field tested before its initial use—no changes were needed afterwards.

**Table 2:** Deductive categories and guiding questions for the observational units.

Deductive category	Guiding question	Transferred observational categories into UTAUT categories
Decision making	For which patients (health condition/disease) is the device used? What factors can be observed in the decision to use it (such as sociocultural, patient comorbidity, consent)?	(Observed) performance
Task	For which tasks is the device used? What unexpected uses can be observed?	
Information	What kind of information does the device provide to the nursing professionals and how do they deal with it? What processes are enabled by the new information?	
Effects on patients	Can changes in patient condition be observed over the course of using the device?	
Satisfaction	In which situations are forms satisfaction, acceptance, criticism or rejection observed towards the device by the nursing professionals and/or patients?	(Observed) effort
Workplace integration	How does the integration of the device take place in the everyday work of the nursing professionals?	
Team communication and team work	How do the nursing professionals talk about the device among themselves and with the patients? Can changes in the teamwork of the nursing staff be recognized using the device?	(Observed) social influence
Expectation towards and reaction from patients	What do nurses expect from patients when using the device? How do patients react to the use of the device?	
Social interaction and substitution	How and in what form does social interaction between nurses and patients take place when using the device? Is there a substitution of nursing activities by the device?	
Resources	How will decisions be made if there are more patients than devices?	(Observed) facilitating conditions
Access to training and technology competence	In which situations do nursing professionals feel confident or insecure when using the device? Are the training formats during implementation (manufacturer training, additional material by the research team) sufficient to use the device?	
Technology characteristics	How can the characteristics of the device in use be described? What is the quality of the product?	

During the field stays, handwritten notes containing summaries, situation descriptions, reflections and ideas were taken. After each observational unit, the observation questions listed in Table 2 were used to structure the writing of open-ended, chronological fieldwork diary entries that reflect observed situations in detail, reproducing dialogues, and characterising people, technologies and situations (32).

(b) Questionnaire-based survey on technology suitability

A technology suitability questionnaire was used to investigate the range of opinions of the nursing professionals. This instrument was used additionally to the observations, because the observation could only incorporate the views of individual employees (who were working at the times observed), rather than obtain the diversity of opinions on a technical product. Therefore, a questionnaire was used that *descriptively* included the respondents’ views on the suitability of the technology for use. Since no meaningful case numbers can be obtained on the project ward, the use of the questionnaire can only be classified as a supplement and contextualization of the qualitative results from the observation. This instrument was developed and used in another research projects (22, 33). The questionnaire was provided to all nurses on the project ward for each implemented product. It contained four sections:

1. Three items on the general use of the product since its introduction (use yes/no, frequency of use and, reasons for not using the technology).
2. *General* questions covering usability, workflow, compatibility, functionality, product quality and patient well-being.
3. Questions *specific* to the technology covering power supply, alarms, screens, mobility, consumables, and reprocessing.
4. Further comments on the device to be entered in free text entries.

In the general and specific sections, the questions were answered using a five-point Likert scale from ’Strongly agree’ to “Do not agree at all”—or “not applicable”.

This is a measurement instrument for technology suitability and not an instrument from the field of technology acceptance/UTAUT research. Therefore, the findings of the suitability survey are classified under the UTAUT category of (observed) effort.

All nurses received the questionnaire for each of the implemented technologies three months after the first deployment of the given technology, either in workshops or in their mailboxes on the ward. In the case of using the individual post box, they were notified at the time of distribution and with a reminder by E-Mail. Due to the long implementation phase in the study, the number of employees on the project ward varied significantly, but on average 22 full-time staff are employed on the ward. However, this number varies, mainly due to staff shortages. As the study design was set up in such a way that only one ward was equipped with technology, the questionnaire could only be used in one setting and comparisons with other wards/settings were not planned in the study design (see also Limitations).

### 2.3 Methods II: initial data analysis

(a) Qualitative data analysis—evaluative qualitative content analysis

The entries from the open field research diaries and the free text entries from the technology suitability survey were analysed using evaluative qualitative content analysis (34). While applying this method, each deductive main code received a set of at least three sub codes for (1) a positive manifestation, (2) a negative manifestation and (3) a neutral or non-evaluative category (35). For example, the main code of “work integration” received the sub codes (1) “smooth integrated”, (2) “problems with integration” or (3) “other”—in this care, a forth sub code for ambivalent observations were also used.

The guiding questions in Table 2 were used to develop the main codes deductively. Only one inductive main code was added for “Expectations of new technologies”. Through this approach, a code system of main codes and sub codes (see Supplementary Data Sheet) were developed that helped to organise the data material and to perform a pre-analysis.

(b) Quantitative data analysis

In the questionnaire-based survey on technology suitability, the answers to the second and third areas (general and specific aspects of the technology) were analysed quantitatively (33). For this purpose, the scores achieved by the technology in each area were first expressed as a percentage of the maximum possible score. These two percentage results were then weighted according to the number of items in each area, and an average, general value were given. If a technology achieved up to 49%, it is considered *unsuitable*; if it achieved 50%–69%, it is rated as *suitable to a limited extent*; and if it achieved 70% or more, the technology is rated as *very suitable*.

### 2.4 Methods III: data consolidation

Quantitative results were compared with the qualitative analysis of the observation data and the free-text entries of the appropriate questionnaires. The data collection and analysis was performed parallel rather than sequentially. We merged and compared the data to identify consistencies, inconsistencies or complementarities (36). The basis for data consolidation and analysis was the UTAUT model, initially presented by Venkatesh in 2003 (16). Four main categories are presented in this model:

▪ “**Performance expectancy** is defined as the degree to which an individual believes that using the system will help him or her to attain gains in job performance.” (ibid.)
▪ “**Effort expectancy** is defined as the degree of ease associated with the use of the system.” (ibid.)
▪ “**Social influence** is defined as the degree to which an individual perceives that important others believe he or she should use the new system.” (ibid.)
▪ “**Facilitating conditions** are defined as the degree to which an individual believes that an organizational and technical infrastructure exists to support use of the system.” (ibid.)

The use of the UTAUT model in our study served two purposes. (1) To arrange and summarise the results along these categories for transparent reporting. (2) To use a mix of qualitative and quantitative findings to explore how much influence each category had on technology use, acceptance, rejection, and adaptation. Therefore, the observation categories from Table 2 were assigned to one of the four main categories, which can be found in the same table. The survey results were assigned to effort expectance based on the construct “Suitability”. In our study, the UTAUT model was used to evaluate observed user behaviour (therefore “Observed Performance” etc.), not used to predict user intention or behaviour. The four mediators’ gender, age, experience, and voluntariness of use (ibid.) will be addressed in the results section if relevant to the reporting.

### 2.5 Ethical considerations

The research project was approved by the Ethics Committee of the Hannover Medical School on the 6th of July, 2018, ID: 7933_Bo_K_2018 (amended 16th of July, 2020). The procedure was reviewed by the hospital's staff council and the clinic data protection officer. The data protection-compliant processing of research results (above all with the aim of protecting study participants) was carried out in accordance with the guidelines of the University Hospital, above all with the help of lockable rooms in the case of hard copies and password-protected drives in the case of digital data. Raw data was only shared with research project participants, and patient care leaders were merely given access to analysed, non-personal, summarized data as required, making re-identification implausible.

(a) Participant observation

The scheme for situationally appropriate privacy expectations was used to identify which individuals in the field should be asked for written consent to observation (37). Written informed consent was obtained from professional nurses observed during their shifts. Before giving their consent, nurses received an introduction to the study's goals and reasons. If possible, participants in the field were informed of the observer's presence, especially to patients when first entering the patient's room (38).

(b) Questionnaire-based survey on technology suitability

Nursing professionals who completed the questionnaire also filled out a written consent form. Sociodemographic data (such as years of professional experience or age) were not collected due to the small size of nursing staff to avoid re-identification.

### 2.6 Quality assurance

For the reporting on methodical decisions and processes in this paper, the COREQ-Checklist was used (39)—all relevant information are provided in the dedicated section of the paper or in Supplementary Image S1.

(a) Participant observation

(1) Key observational findings on the impact of technology implementation were presented, discussed and again documented in dedicated validation workshops as a form of “respondent validation” (40). (2) The research team reviewed and discussed result plausibility and implications at periodic meetings internally and in external research workshops. (3) Parts of the results have already been presented regarding individual technologies and selective research questions at conferences (41, 42). (4) The observed nurses were offered to read the diary entries after completing them; however, no participant used this offer. Coding and consolidation of the data material was performed independently by two authors (RK & DK) and then compared. Intercoder reliability was not numerically calculated.

(b) Questionnaire-based survey on technology suitability

Two independent data entries were made to ensure no errors occurred during the transfer. The main results of this survey were presented in the validation workshops to the nurses mentioned above. All results from the questionnaire survey were presented, interpreted and discussed within the interdisciplinary team of the research project.

## 3 Results

### 3.1 Participant observation

Observations began in July 2020. New technology has been explored in 23 observation units, representing 38.5 h of observation time. The author RK conducted all observations. The average time of an observation unit is two hours. These observations resulted in 132 pages of field research diary entries. Member validation workshops were protocolled. The results of these data collections are summarised in this chapter. In the course of the participant observations, fifteen nurses could be accompanied on their shift. All but one of the nurses responded positively to be observed—the person who did not wish to participate has been omitted from all observational descriptions.

Table 3 provides an overview of the observations’ results on the implemented technologies reported by UTAUT's main categories. The boxes in the table are marked with colours and indicate whether the observation results for the corresponding UTAUT category are characterised as favourable for the use of the technology (green box), adverse and unfavourable (red box), or both positive and negative and thus ambivalent (yellow box). Categories with no effect are left blank.

**Table 3:** Results of ethnographic observations analysed by UTAUT-categories.

UTAUT categories	(Observed) performance	(Observed) effort	(Observed) social influence	(Observed) facilitating conditions
	*Note: The colour coding in the fields indicates whether the corresponding factor bundle is predominantly positive (green), negative (red) ambivalent (yellow) or has no influence on technology acceptance (not filled).*			
Mobilisation mattress	Decision for using based on risk assessment and professional intuition and depends on: Diagnosis, especially postoperative patients with pain during positioning Time of the day (only in the night or 24 h) General patient condition (few proper motion, high risk for pressure ulcer) Perceived overall reduction of pressure ulcer frequency from nurses perspective	Reliable system, performs work “*in background*” that relieved time resources for other tasks Perceived reduction of back pain for nurses Became part of routine practice in course of research project	Collaboration with colleagues during decision making, actual use is done in individual work routine Patients usually get informed by nurses on function of the mattress Patients report better sleep, few patients report form of “motion sickness”, most get used to the sensation. Nurses perceive improvement of relationship to patients due to better sleep Substitution or reduction of social interaction not observed	High demand by nurses led to an increase in stock to sufficient number of systems Upper body elevation up to 50 degree is optimal (former 30 degree version also tested; led to additional problems) No lack of competences to use system observed, calibration is perceived to be simple
Fall prevention system	Use in patients prone to falls due to individual medical condition (e. G. dementia) Mixed reports on whether technology actually reduces falls Nurses report false alarms and see technology as unreliable Use even when it is unreliable	Dissatisfaction based on time-consuming installation, false alarms and frequent defects that require external repair	Preparation of the use of the systems (installation in the patient bed) in teams, especially in the night shift Team work performed while users take care of small repairs of malfunctioning systems in teams No change in social interaction between nursing professional and patients due to technology observed	Resource scarcity depends on dysfunctional systems Nurses report false alarms or not activating alarms in case of real bed-exit attempt Insecurity about correct installation and use of the system even after manufacturer trainings
Audio-haptic sound pillow	Mainly used for patients with agitation, challenging behavior and pain Positive response from patients (calming, activity/interaction with the technology) Patients lose interest after few days based on monotony of the melody and deactivation of the technology after inactivity Use during night times limited to patients in single bed room	Used to increase patient adherence to nursing intervention Used as an additional means of calming patients down Nurses have to reactivate pillow due to automatic stand-by Technology allows nurses to leave patients unattended for short periods of time	No relevant team work or communication observed Nurses try to present device in an interesting way for patients If patients will not respond to verbal presentation, they present it non-verbally Some patients are not interested, others are highly interested	No resource problem observed The cover can be disinfected with wipe disinfection Every new employee receives a short introduction into the technology, no competence related problems observed
Special projector	Patient-dependent decision-making process made by nurses (who, where, when, which module?) Positive response from patients Used in patients with agitation, challenging behavior Outcome depends on desired goal: Calming modules at night when patients need to rest Activating methods during the day with agitated patients	Ease of nursing care, especially for time and effort to be spent on patients with challenging behaviors Nurses are satisfied with use and the outcomes Nurses perceive (de-) installation and use as easy, technology became part of routine practice	No relevant team work or communication observed Nurses perceive technology to have a positive effect on nurse-patient relationship Some patients’ reaction is negative on particular modules, nurses have to respond to patient reaction and chose suiting module Fellow patients could perceive technology (sound, light) as annoying	No resource problem observed Technology offers a variety of modules with different visualizations, sounds and operating modes Every new employee receives a short introduction into the technology, no competence related problems observed
Interactive therapy ball	Primary use for patients with mild states of confusion or dementia, but who are still oriented Supposed to lead to an improvement in the general mental state of patients; however, the technology has been used too rarely in practice, so that no observations could be made on effects	Nurses see positive and easing benefits of the technology, but cannot use it in everyday practice (familiarization and use is too time-consuming). No sustained use was observed, tested on few occasions, especially in educational situations	Few uses are observed in training situations, trainees have more time to interact with patients Potential for positive influence on nurse-patient relationship is suspected by nurses, but work conditions prevent regular use	Time is a limiting factor Functional spectrum is complex and menu navigation needs time for using Nurses workload is too high for using the technology Operation via remote control, nurses wish for voice control
Patient-Nurse communication app	Use depends on nurses’ assessment of whether patients can operate an app Used to remind nurses about upcoming tasks, events or to prioritize patient requests Use of the technology can reduce redundant path and/or reduce noise from acoustic nurses call sign However, nurses doubt performance for work after few weeks of use Empirically app is mostly used for nursing care (not service) related requests	Nurses are concerned about what app suggests to patients about nature of nursing care (*nursing care as a service instead of a health care profession*) Some nurses refuse to work with additional (smart) phone Doubt that redundant path reduction has a relevant ease in daily walking distance Nurses criticise that nursing care is more than the reaction to articulated patient wishes but an interaction that allows to grasp patients’ needs	Successful use of app requires continued use of app between shifts, which is rarely observed Ambivalent reaction by patients that used app: most find it helpful and easy to use, some do not see the benefit for patients (but recognise benefits for nurses) Faster and more transparent communication between nurses and patients was observed Nurses expect patients to use app primarily for nursing (not service) related requests	A smartphone for each section of the ward is provided for using the tec. The patients have to use their own smartphone, download app and register via specific code High technology competence required for patients and nurses, merely fraction of patients on ward are suitable
Tracking system	[Contrary to initial nurses intention to use the technology, tracking turned out to be not helpful] Nurses report that when they are searching for a product, they ask colleagues or have seen it during their daily activities No effects on patients observed	Low satisfaction due to lack of relevance No workplace integration into routine practice (although full technical implementation), because no practical relevance for work Nurses suggest that technology would be helpful if it could be applied to the entire clinic	Communication with other colleagues about where (technological) products are makes the technology useless No effects were observed regarding the social interaction and substitution with patients	Wi-Fi and tablet to use system was installed and is functioning, no technical issues are observed The system could find products which are provided with a Bluetooth-tracker

#### 3.1.1 (Observed) performance

The mobilisation mattress and the special projector provide a positively perceived performance from the point of view of nurses and patients. The mobilising mattress is frequently used on the project ward. The individual risk assessment for the development of pressure ulcers is not the sole deciding factor for whom, when and how the system is used:

*While we walk to the next patient room, the nurse says that the mattress: “almost does not matter during the day”. She explains that many patients lying in the systems require intensive care anyway, such as patients with incontinence pads that need to be changed regularly. For these patients the mattress is advantageous at night because using the system helps position patients less frequently, and one needs to wake them up less often. […] Patients who suffer from much pain are an exception: People who have suffered trauma will experience less pain due to the system’s movement. (Field Research Diary_Mobilization mattress-BE04, p. 2)*

Other nurses run the system on all patients and use the system’s pause function instead to perform interventions. The special projector is primarily used in patients with dementia or agitated behaviour in two different ways: One is to calm nervous patients and address challenging behaviour. The other way is by reactivating apathetic patients, in whom the use of the projector activates memories. According to the nurses, both ways can help improve care of these patients, making care delivery easier. The decision-making is biography-based or stems from getting to know the patients' behaviour. The special projector and the mobilisation mattress are often used together.

Technologies that have shown **mixed and thus ambivalent performance** in the ward are observed for the sound pillow, the fall prevention system and the communication app. The sound pillow functions according to its intended purpose, as the following situation description shows:

*A mask for inhalation is placed on the patient’s face—the sound pillow lies on his chest. The nurse seems surprised and says this was not easy in the last few days because the patient kept pulling the mask off his face. The patient now seems sleepy—about 2 min pass. The patient gets quieter and finally almost falls asleep. The patient seems so calm that the nurse wants to leave him alone to return in 15 min. (Field Research Diary_inmu-BE01, p. 28)*

Most patients lose interest in the technology after a few days due to its repetitive sound. Therefore, an actual benefit is limited to a time range that is shorter than the patient's hospital stay. For the communication app, situations are observed where patients send their requirements, nurses read them and can react according to the current workflow, for instance to take medication with them to the room. However, the app is rarely used since some nurses question whether the app makes a difference in everyday work. For the fall prevention system, it is observed that nurses respond immediately to a bed exit. However, the perceived performance of the system is low as users report frequent false alarms or missing alarms, resulting in low system confidence.

*The nurse reports that the system was running overnight but did not activate even when the patient already stood in the room. (Field research diary_SaSe-BE01, pos. 9)*

The perceived performance of the system for equipment tracking is low. The system proves to have no technical problems in practical tests, so non-use initiates from a lack of practical relevance for the users. Communication and teamwork among nursing colleagues to find equipment is easier to realise according to nurses.

The interactive therapy ball is rated as **neutral** regarding its perceived performance because users cannot operate the technology as intended (this will be explained in detail below).

#### 3.1.2 (Observed) effort

Nurses perceive the mobilising mattress and the special projector as easy to use. Both systems are perceived to be reliable and supportive of work processes, saving effort on time-consuming tasks and helping cope with work process-related requirements. They are perceived as being easy to install and are considered part of daily work routines. The devices do not need to be operated constantly but can be used partly autonomously (in the background), as the following entry illustrates:

*While documenting, the nurse said, “On days like today, the system is worth its weight in gold.” I asked what she meant by that. She explained that with the system, she could sit at the PC for as long to document. Repositioning the patient to prevent pressure ulcers would require her to interrupt her current activity regularly. I asked her if she was confident the system was doing a good job in the background. She confirmed this and said that it was a great relief. (Field Research Diary_Mobilization mattress-BE04, p. 3)*

The nurses repeatedly emphasise that using the mattress and the projector does not mean patients are left alone for long periods and interactions between nurses and patients are not reduced. Instead, it changes the nature of the interaction by removing specific tasks perceived as unpleasant, such as positioning patients.

Positive effects and ease of use are identified with the sound pillow and the interactive therapy ball, but to a limited extent. Nurses evaluate that the sound pillow has a calming effect on patients. This calming effect, in turn, directly influences patient adherence to specific therapeutic measures and makes it easier for patients to cope with difficult emotions or pain. However, many patients lose interest in the technology after a few days of use. A patient can use the pillow without the constant supervision of a caregiver. For the therapy ball—that in contrast needs the permanent presence of a caregiver –, no sustainable use can be observed. Nurses and trainees use this device in a few instances and have positive experiences, but could not use it in everyday practice due to a lack of time. Therefore the technology's easing effect could not be realised under the given work organisation.

In the case of the communication app, the tracking and fall prevention systems, findings suggest that the devices require additional effort for **little to no benefit**. Nurses do not see any practical benefit for the tracking system. However, an expansion within the entire hospital could be beneficial. For the communication app, some nurses find the additional smartphone impractical in everyday practice, because they are not always within reach or their pocket are already packed with other items. While the fall prevention system is used in practice, nurses mention frequent technical problems, most of the users see the product as having little overall benefit:

*The nurse currently has a patient lying in the fall prevention system. This patient has not tried to get up recently, but the system has been alarming at regular intervals. This makes the system unusable; she adds “You make an effort to set it up, and then it does not even work”. (Sound pillow_Fragment 01)*

#### 3.1.3 (Observed) social influence

Four of the introduced technologies **positively influenced the interaction** between nurses and patients. The three technologies for patients with challenging behaviour performed similarly in this area. Teamwork is performed merely when a nurse seeks advice from colleagues on selecting suitable patients. After that, the nurses work with the technology without further cooperation. The technologies have a positive impact on nurse-patient interactions, as the following two research notes demonstrate:

*For the special projector, the nurse likes the forest-walk module. She had a patient with dementia who used this module and, while watching, tried to find out where the shots might have been taken. (Special projector_Fragment_01, pos. 13)*

*The nurse had a night shift, and a patient could not find rest and walked around the room for several hours. She gave him the sound pillow. After that, the patient slept soundly for hours. (Field Research Diary_Communication app _BE02, Pos. 5)*

Nurses also emphasise a module that displays a night sky with shining stars that is selected for patients to fall asleep at night. Nurses say that the calming and activating use of the sound pillow and special projector enable easier interaction with these patients and fewer challenging situations and conflicts. While using the mobilizing mattress, patients find better sleep than those who have to be woken several times during the night for positioning. Nurses describe that sleep improvement also improves relationships with patients.

An **ambivalent influence** of technologies on the social interaction of users is found in the fall prevention system and communication app. The fall prevention system does not directly affect the relationship between nurses and patients. Although patients are repeatedly surprised that nurses quickly enter the room when they try to stand up. Repeated technical problems, malfunctioning components, or the system installation lead to negatively perceived collaboration between nurses. The mediator category *voluntariness of use* explains why the product is frequently used on the project station. It seems plausible that the nursing supervisor requires the system to be used for liability. This factor is part of why the device is frequently used, but the overall satisfaction is low. While the Patient-nurse communication app is used, some patients particularly emphasising the benefit of additional information, such as how long they must wait for a response. The following conversation is observed between a nurse and a patient:

*A feature of the app that both consider useful is task prioritisation. Both talked about how it can make sense if you know that a request such as “close the window” occurs in one room and “severe pain” in another. Both agree that it is good to process first the pain and then the window request. (Field Research Diary_Communication app_BE01, Pos. 21)*

For other patients, the app has no advantage because the waiting time does not change. In addition, nurses are cautious in selecting the appropriate patient to use the app. They are concerned about low-skilled patients who send requests by accident. Others fear that the app suggests professional nursing to the patients as a (hotel) service.

None of the technologies introduced have an **overall negative impact** on the users’ social relationships. Regarding the tracking system, nurses find no support for the technology because communication between colleagues is more effective. Therefore, the social factor is still a robust explanatory category for non-use of technology.

#### 3.1.4 (Observed) facilitating conditions

For the special projector, the sound pillow and the mobilisation mattress, **sufficient resources** for using the technologies—like technical infrastructure—are provided. Therefore, no conflicts about too few devices are found for these technologies. All nurses receive detailed training for these devices. The mobilising mattress had a problematic feature at the beginning that deactivated the system if the patient raised the head of the bed by more than 30 degrees. This often leads to unintended deactivation by patients. After consultation with the manufacturer, the limit was elevated to 50 degrees. Since this update, nurses reporte fewer problems. The sound pillow and the special projector are easy to integrate into existing facilities. All three systems can be cleaned with the regular disinfectant on the ward and no severe technical malfunctions are reported.

For the tracking system and the communication app, the findings indicate that facilitating conditions have **both positive and negative influences on** the use of technology. Although nurses receive training on how the technologies work, in practice, there are regular uncertainties about use. The tracking system and the communication app run mostly without technical problems. The wi-fi coverage on the station is sufficient to provide both services most of the time. In a few instances, there have been examples of the tracking system showing the wrong location of the tracked equipment:

*The nurse says there was an incorrect location in the system for an electronic rail. He says that it was indicated in a different room than it was. […].The access points are installed too close to each other […].’ (Field Research Diary_Communication app_BE02, item 31)*

Nurses suspects that messages from the communication app sometimes do not get through in real-time. For patients, there are currently no input devices for the app on the ward so patients must bring their smartphones to use the app. Nurses must explain the downloading and functioning to patients if they require assistance. The nurses receive this point critically since they have no time to train patients. For this reason, nurses select patients in particular by anticipating their technical abilities and patients must be motivated to use the app.

The technical and organisational conditions are **limiting factors** for the therapy ball and the fall prevention system:

*“I have no time for [the therapy ball]. An everyday companion would have time.” “I dealt with it once and then I knew how it worked, but now I have already forgotten about it.” (Protocol of member validation meeting, June 2023)*

Hence, the device's menu navigation is seen as complicated. The nurses would like to use the therapy ball and would enjoy working with it but do not see the time for this. The fall prevention system exhibits system errors and false or outstanding alarms that hinder its use. Caregivers repeatedly report that the device's correct installation and operation is complicated, resulting in uncertainties.

### 3.2 Technology suitability from nurses’ perspective

The survey on technology suitability could be conducted on all technologies. The results can be found in Table 4.

**Table 4:** Results of the suitability survey by nurses that used implemented technology.

Technology	No. of participants	No. of participants that used the technology*	Frequency of use				Reasons for not using the technology**	General suitability***		
			Daily	Weekly	Monthly	Less often		Low	Medium	High
Mobilisation mattress	12	12	1	4	5	1	[None]	1	2	9
Fall prevention system	10	8	1	4	2	1	No opportunity (1) Does not help me with my work (1) Workflow is faster without (1) The handling was not clear to me (2)	3	2	3
Audio-haptic sound pillow	9	7	0	2	7	0	No opportunity (2)	0	0	9
Special projector	5	5	n. a.	n. a.	0	0	5			
Interactive therapy ball	6	4	0	0	1	3	Workflow is faster without (1) Setup/handling not practical (1) Unaware of the technology (1)	0	3	1
Patient-Nurse communication app	9	3	0	1	1	1	No opportunity (3) Does not help me with my work (3) Workflow is faster without (1)	0	1	2
Tracking system	6	3	0	0	0	2	Does not help me with my work (3) Workflow is faster without (1) The handling was not clear to me (1)	1	1	1
Free text entries										
Mobilisation mattress	One person noticing that battery operation would be more practical, especially when patients move within the clinic. Another person reports that it is easy to forget the reactivation of the mattress after pausing it during meals. Five people comment that the automatic deactivation of the system is impractical.									
Fall prevention system	One person rates the device positively, and one mentioned “constant false alarms”. Another notices that it is impractical when the system can only be turned on and off directly at the bed and that it is difficult to see whether the system is activated.									
Patient-Nurse communication app	One participant notes that he or she does not want to use the app. Another person says that he or she likes the app but had no opportunity to use it.									

* The difference between this number and the number of participants represents the number of people who filled in the questionnaire but have not (yet) used the product.

** Number (in brackets) indicates the number of participants who have selected the corresponding item.

*** Numbers indicate the calculated item of general suitability per technology (e. G. a 9 for “high” indicates that nine participants rate the general usability of the technology as high). For example, technology can achieve 56 points in the general area, but it is assessed with 30 points or 53%. In the specific area, 32 points can be achieved, and 30 points are awarded, giving a score of 93%. When weighted, these sub-scores give a technology suitability of 68%.

n. a. Due to an error in the preparation of the questionnaire, no feedback on frequency of use could be collected for the special projector, which is why the data is missing from the table.

The number of participants varies because the average number of nurses working on the project ward varied during the research project and not all nurses participated in the survey. Similarly, not every technology was used by all employees; in particular, temporary workers often stated that they had not used the technology due to short training periods on the ward. Other people also stated informally that they did not have time to complete the questionnaires during daily work. For these reasons, the number of participants in the surveys varied from five to twelve employees (as described above, an average of twenty-two people work on the ward at full-time employment).

The comparison between the observational results and the standardised survey shows a coherent picture. The technologies are described as easing and beneficial (mobilisation mattress and special projector) are also evaluated positively. In contrast, the ambivalent (sound pillow and therapy ball) and unfavourable technologies (fall prevention system, communication app, and tracking) receive mixed evaluations. The frequency of use is also consistent to qualitative results; The technology that stood out in the observations as accepted and used received a higher frequency in the survey, like for the mobilization mattress (used daily or multiple times a week).

### 3.3 Summary of results

A summary of results can be found in Table 5. The results are consistent with the observational data and the survey on technology suitability.

**Table 5:** Summary of results according to use and acceptance.

	Technology accepted	Technology not accepted
Technology used	The *Mobilizing mattress*, the *Special projector* and *the Sound pillow* are accepted and regularly used. The technologies are positively classified in terms of usability and either scored positively in all four UTAUT categories or, in the case of the sound pillow, in two of the four outcome categories. Thus, the social environment and the facilitating conditions influence all three products’ acceptance. The products received predominantly positive feedback regarding perceived performance and ease of use, with limitations for the sound pillow that was accompanied by conflicts.	The *Fall prevention system* is used regularly on the ward. However, its acceptance is low, and nurses view the system negatively. In their experience, it regularly indicates false alarms, does not register attempts of bed-exit and is complicated to set up. Nevertheless, the fact that the system is used can be explained by the mediator variable “Voluntariness of Use”. Several nurses noted that they would stop using the technology as soon as a better alternative is available.
Technology not used	The *Interactive therapy ball* is not used on the ward, because workload and lack of time makes it hard to use. However, the nursing professionals’ general attitude towards the technology is high. The nurses would like to use the technology in their everyday work, and they would also see a benefit but cannot actualise it.	The *Patient-Nurse communication app* and the *Tracking system* are neither regularly used on the ward nor do nurses see benefits from these technologies. Although expectations were initially high for both products to have a meaningful impact on the ward—and in technical terms, the systems function well—the nurses were unable to identify any meaningful forms of use for the technologies, even after several months of implementation.

## 4 Discussion

### 4.1 How accepted and not used technology can (not so easily) be distinguished

The observation results of the study were summarised along the four main UTAUT categories and the positive, negative or ambivalent influence on technology acceptance per category per product was identified. These results can be compared with the technology suitability survey, which provides information on the assessment of nurses and the frequency of use. But how to answer which technology was used, which was not and how to distinguish the influencing factors as enabling or hindering factors?

The conclusion of whether a technology was accepted or rejected cannot be based solely on the positive, negative or ambivalent results of individual UTAUT categories, because this would confuse the phenomena to be explained (explanandum) with what it is explained by (explanans) (43). Until the outcome of the implementation process is uncertain, classifying the influential factors from the UTAUT categories in the implementation process is unattainable. At this point, frequency of use could be utilised as a proxy for general product acceptance but little data could be collected on this. In addition, a technology may be highly accepted even though it does not need to be used regularly.

To solve this problem, we use ethnographic sensibility. This term refers to a feeling or impression towards the *ethnographic*, i.e., the lived and experienced reality in the research field, about its “complexity, contradictions, possibilities, and grounds [for the observed] cultural group” [(44), see also (45)]. This sensitivity was gained by the observer over years of field research activity and enables knowledge about the users’ general attitude towards the technologies, which was needed to situate the results eventually.

The above presented summary of four result categories leads to the follow consequences:

(1) A predominantly positive influence of the technology in terms of the UTAUT categories (see Table 3, green fields) strongly indicates that the technology is used and accepted.
(2) Use and acceptance cannot be equated. The fall prevention technology—acceptance low, but regularly used—and the interactive therapy ball—high acceptance, but not used—shows that these outcomes do not have to exist simultaneously.
(3) The occurrence of more than one origin of negative and ambivalent influence of the technology (see Table 3, red and yellow fields) strongly indicates that the technology is not accepted or used regularly.
(4) The main categories of the UTAUT model can be a strong indicator for explaining technology acceptance. However, they should be distinct from explanatory factors because factors like perceived usefulness or ease of use occur within broader socio-technical constellations and contexts of actualised technology use (46). Explanatory power unfolds with an understanding of the use context. This context was approached in our study by using ethnographic sensitivity.

To conclude the four consequences, it takes more than adding variables to predict user acceptance towards technologies. Instead, acceptance emerges as the result of complex socio-technical arrangements in which users must convince themselves of the benefit of technology for their actions by constantly trying, failing and succeeding.

### 4.2 Intention to use technology must be stabilized by experimenting

Some expectations users set regarding a technology's usefulness were not met after implementation. The intention to use technology indicates a necessary *curiosity* that motivated the start of technology use. However, this is *no guarantee* that a sustainable technology acceptance will occur. Users take cautious first steps in using novel technologies when familiarity with and skill to use technology still needs to be established. In this initial, critical experimentation phase, users renegotiated attitudes toward the technologies through positive or negative experiences.

On the one hand, unanticipated adverse effects—such as frequent false alarms—could change a high expectation into scepticism or reservation (5). On the other hand, surprising or hoped-for effects that turn out to be true could result in positive attitudes among users. This was frequently observed, for instance, when nurses asked whether they could be supplied with more system mattress, sound pillows or special projectors to cover demands.

Different users face the introduction of technologies with different skills and prior experience and with varying degrees of optimism or scepticism. Age and experience as mediator variables in the UTAUT model provided a valuable orientation for our analysis. However, introducing a helpful technology can transform existing work conditions, changing how a work field and a social reality functions (47). While the different preconditions among users may provide clues to different levels of acceptance and rejection, a helpful technology can *change* these preconditions among users [for the case of generalised distrust among nurses towards technology, see (48)]. Thus, it is more plausible to assume that a rejected technology does not bring any actual benefit instead of assuming a primordial attitude of rejection among users who would not give valuable technologies a chance (and vice versa).

### 4.3 Acceptance may not be sufficient

Our results show that four technologies—the mobilising mattress, the special projector, the sound pillow and the fall prevention system—offer a way to mitigate the high demands of a professional nursing work environment that is increasingly characterised by staff shortages and a growing number of multi-morbid patients. The other three technologies—the tracking system, the communication app and the therapy call—could not meet these demands. From an acceptance perspective, this can be understood as fulfilled or unfulfilled device performance expectations.

Alternatively, these results can also be explained by the fact that the successful technologies can be operated in a background mode. A “background relation” between a technology and a user can be explained by a device that the user does not continuously operate—i.e., it works in the background—but nonetheless shapes the environment and the user's experience (49). A background technology does its work without the need for permanent operation. Solely in case of a malfunction, users are reminded about its importance and have to act in an effort to repair it—an example would be an air conditioning system. It is opposed to a technology that requires the user's constant input.

The features of the mobilising mattress, the fall prevention system, the special projector and sound pillow can be utilised without constant manipulation and nurses’ presence, which makes them handy on stressful workdays. The communication app, the tracking system and the interactive therapy ball cannot be used similarly. For the therapy ball, for instance, nurses emphasised that the permanent input needed for the system’s operation is the reason they were not using the system after all.

However, when viewed from the perspective of patients, the background characteristic is problematic. After all, this implies that patients receive parts of care by technology. For instance, in the case of the mobilising mattress, re-positioning a patient to prevent pressure ulcers is not executed by a human but by the technical system, changing the caregivers’ task from an active part of *doing the reposition* to the *passive part or controlling* the technologies output. At the same time, nursing action as interaction work consists of more components than executing a nursing care action (50). As such, it also consists of emotional and sentimental labour, in which the nurse can recognise the patient's needs through interaction and communication with them and then react based on these encounter (ibid.).

The mere adoption of tasks by technology is no evidence of less social interaction between professionals and patients—also we did not collect data on contact times. However, technology that is successful *because* it is usable in the background may eventually reduce opportunities for interaction. The evidence of successfully implemented technology that supports nurses in managing their increasingly demanding workday under staff shortages might indicate that technology is accepted because it enables them to continue working under problematic conditions. Implementing technology may therefore reinforces problematic developments (more missing human resources) rather than questioning it. For this reason, looking purely at acceptance as a measure of successful use of technology in care may fall short. Instead, the potential change in the levels of interaction and resonance between nurses and patients caused by technology use would be a possible outcome for qualifying technology acceptance (51).

### 4.4 Limitations

 (1) Effects were primarily perceived by the observers and the perspective of the observed is only described from “outside” No interviews were conducted—at the time of reporting—to involve the individual perspectives. However, at least in their validity, results could be discussed and confirmed with nursing professionals.
 (2) The results from the questionnaire are subject to substantial limitations since participation varies to a high degree.
 (3) The study's argument is based on the assumption that, due to a participatory introduction process, only those technologies found their way onto the ward that the nurses also desired. However, it was impossible to verify whether this assumption could be applied to all nurses.
 (4) The narrow patient population on the project ward influenced the selection and the use of the technologies. In the example of the communication app, little benefit for the nurses could be seen because too few patients had the skills to use an app. In this respect, the (qualitative) transferability of the results to other clinical settings is limited.
 (5) Patients’ perspective is marginally represented in this paper because patients in the case of the project station are mostly passive technology users or beneficiaries and have no direct experience with the devices or cannot verbalise this, for example, due to dementia.
 (6) All costs associated with the acquisition, operation, malfunction and repair of the technologies were covered by project funds. Therefore, the transfer of interpretations to other health care settings is restricted, particularly in terms of (sufficient) resources. In other health care institutions, for instance, budget restrictions could trigger negative usage effects. The German healthcare system continues to lack sustainable, cross-setting and comprehensive solutions for financing innovative technologies. The same applies to the *amount of work* required for maintenance, servicing and in the event of malfunctions and repairs. In non-research settings, this must be carried out by employees and can have additional, negative consequences for the use of innovative technologies.
 (7) The decision to equip one ward with technology in the course of the implementation activities was designed to achieve a summative (qualitative) effect through the combination of different technology approaches. Although this decision enables the investigation of interaction of technologies in one setting, it disqualifies cross-setting comparisons of the effect of technology.
 (8) The seven selected systems are not integrated into existing hospital IT-systems—either because they have their own technical infrastructure (e.g., the app for communication or the tracking system) or because they do not need to communicate with other systems. This limits the implications of the study through the selection of technologies, as it was not possible to make any statements about the usage effects of interoperable systems and their advantages and disadvantages. The decision in favour of isolated solutions was made due to closed hospital IT systems that did not allow the installation of integrated systems.
 (9) A direct calculation of the frequency of technology use (e.g., how often nurses used technologies or on how many patients the technologies were used on) was not achieved. The main reason for this is that it would only have been possible to count on site, but the research team could have not been permanently on the side and the nursing staff refused to document the frequency of use due to a lack of time. For this reason, the feedback from the observation and validation workshops and the corresponding item in the written survey were used. Although these are merely indications and no hard figures, they are not of primary interest in the context of the research question, as the aim is to identify qualitative reasons for use and non-use.
(10) The influence of the mediator variable gender cannot be systematically evaluated in this study, as most employees on the ward are female. However, a direct comparison of the data with the few (three to four) male nurses does not reveal any relevant differences in use patterns or attitudes toward technology.

## 5 Conclusion

In the research project “*Centre for Implementing Nursing Care Innovations*”, we explored the implementation and use of seven technologies intended to support nursing care in a hospital-based trauma surgery ward. The question was investigated which of these technologies are used and accepted or not used and rejected and which factors are responsible for this.

A Mobilising mattress, a Sound pillow and a Special projector were accepted and used, whereas a Fall prevention system was used but technology acceptance among nurses were low do to a perceived low technology quality. A system to track work equipment and an communication app for patients and nurses were neither used nor accepted because users were not able to find a suitable use case, whereas an Interactive therapy ball was accepted among nurses but work condition prevented its application.

The following practical implications can be drawn:

▪ The finding indicates that acceptance of a technology should not be confused with the use of a technology. The technology might be used but acceptance is low, if, for instance, the use of the product is expected as a work obligation. In this case, users may find the technology not helpful and sustainable transfer of technology in routine practice is weak. Likewise, a technology may be accepted and users would like to transfer it into routine practice but circumstances hinder its use. In this case, an institution should facilitate chancing working conditions if the technology is desired.
▪ The categories of performance, effort, social influence, and facilitating conditions provide a practical analytical approach to identifying acceptance or rejection factors. However, they merely provide indications of actual usage and acceptance patterns. The analysis and thus the understanding of the context of technology application itself is necessary in order to be able to classify and qualify overall acceptance.
▪ Experimenting with technology stabilises the intention-to-use into a sustainable use of technology that is adapted to the application context. If users do not find a way to transform this intention into a helpful benefit or if negative unintended or unanticipated consequences emerge, acceptance of the technology remains low. Intention-to-use is not a solid characteristic among users. Users should be given the opportunity to experiment with a new technology to stabilize an intention to use.
▪ In the practical field of nursing, the outcome of technology acceptance should not be viewed simply as the realised use of technology but rather against the background of whether nursing tasks and goals have been achieved through the use and acceptance of technology, such as the improvement of emotional, sentimental and interactive work between nurses and patients.

## Data Availability

The raw data supporting the conclusions of this article will be made available by the authors, without undue reservation.
