# Supplementary figures and images for: Beyond technology acceptance—a focused ethnography on the implementation, acceptance and use of new nursing technology in a German hospital
Source: Front Digit Health. 2024 Apr 25;6:1330988. doi: 10.3389/fdgth.2024.1330988 (PMC11085260; doi:10.3389/fdgth.2024.1330988)

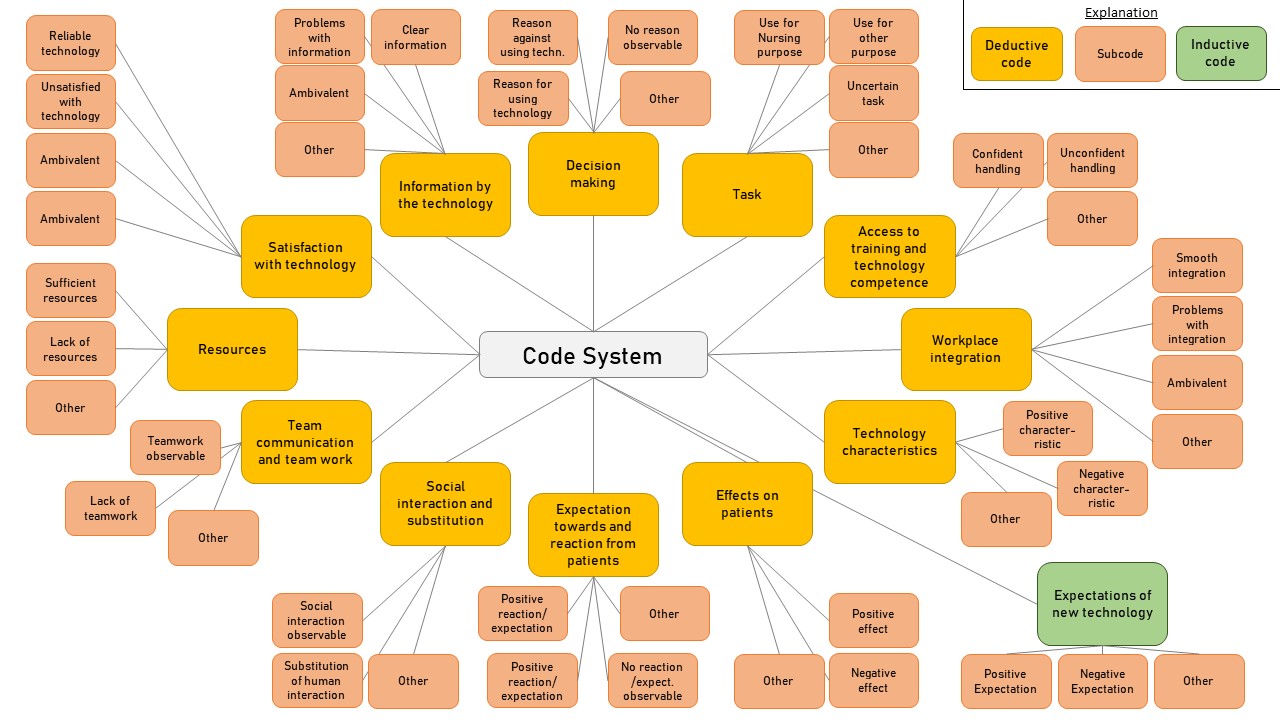

Supplement: Supplementary file 2 [file Image1.jpeg]
